# Persistent resampling of external information despite 25 repetitions of the same visual search templates

**DOI:** 10.3758/s13414-024-02953-z

**Published:** 2024-09-16

**Authors:** Alex J. Hoogerbrugge, Christoph Strauch, Tanja C. W. Nijboer, Stefan Van der Stigchel

**Affiliations:** Experimental Psychology, Helmholtz Institute, Utrecht University, Heidelberglaan 1, 3584 CS Utrecht, The Netherlands

**Keywords:** Visual search, Visual working memory, Long-term memory multi-template search, Eye tracking

## Abstract

We commonly load visual working memory minimally when to-be-remembered information remains available in the external world. In visual search, this is characterised by participants frequently resampling previously encoded templates, which helps minimize cognitive effort and improves task performance. If all search templates have been rehearsed many times, they should become strongly represented in memory, possibly eliminating the benefit of reinspections. To test whether repetition indeed leads to less resampling, participants searched for sets of 1, 2, and 4 continuously available search templates. Critically, each unique set of templates was repeated 25 trials consecutively. Although the number of inspections and inspection durations initially decreased strongly when a template set was repeated, behaviour largely stabilised between the tenth and last repetition: Participants kept resampling templates frequently. In Experiment 2, participants performed the same task, but templates became unavailable after 15 repetitions. Strikingly, accuracy remained high even when templates could not be inspected, suggesting that resampling was not strictly necessary in later repetitions. We further show that seemingly ’excessive’ resampling behaviour had no direct within-trial benefit to speed nor accuracy, and did not improve performance on long-term memory tests. Rather, we argue that resampling was partially used to boost metacognitive confidence regarding memory representations. As such, eliminating the benefit of minimizing working memory load does not eliminate the persistence with which we sample information from the external world – although the underlying reason for resampling behaviour may be different.

**Supplementary Information:**

The online version contains supplementary material available at 10.3758/s13414-024-02953-z.

## Introduction

Visual search is one of the most common tasks that we perform throughout the day (Wolfe, 2010). Frequently, we search for our friend in a crowd, for a symbol on our phone’s keyboard, or for a screw while assembling furniture. Although these tasks may seem trivial at first, one visual search task requires the completion of several subtasks. For example, we must first know what we are searching for; we won’t be able to find the screw that we need if we don’t know what it looks like. To this end, we encode the search target (e.g., memorize a picture of the screw from an instruction manual), and maintain a template of its appearance in visual working memory (VWM). Alternatively, if we have done this task before, we can remobilize existing long-term memory (LTM) representations of our target (instead of visually sampling from the instruction manual) and hold it in VWM. This VWM template then helps us guide search towards possibly relevant locations, and allows us to decide at each of those locations whether what we see matches our internally represented template, or is something else (Olivers & Eimer, 2011; Palmer et al., 2000; Wolfe, 2021).

It is evident that VWM is an essential component within the guided visual search process, although *when* and *how much* VWM is loaded depends on the task constraints, and the limitations (or facilitation) provided by the environment. Specifically, eye movements are relatively effortless (Koevoet et al., 2023b; Theeuwes, 2012; Theeuwes et al., 1998), which is why participants generally prefer to make just-in-time eye movements towards relevant external information rather than to load up and maintain ’effortful’ VWM representations (Kahneman, 1973; O’Regan, 1992; Van der Stigchel, 2020). This preference has been robustly shown across various tasks, with participants tending to minimize the number of items that they encode into memory (Böing et al., 2023; Draschkow et al., 2021; Droll et al., 2005; Hayhoe et al., 2003; Hoogerbrugge et al., 2023; Koevoet et al., 2023a; Melnik et al., 2018; Risko & Dunn, 2015; Risko & Gilbert, 2016; Sahakian et al., 2023; Somai et al., 2020; Triesch et al., 2003). Furthermore, participants often reinspect previously encoded items, suggesting that they also encode less elaborately, and prefer to use external information to refresh internal representations instead (Ballard et al., 1995; Hoogerbrugge et al., 2023; Koevoet et al., 2023a; Sahakian et al., 2023).

This minimization of VWM load also occurs in visual search tasks when templates can be resampled throughout. In those cases, participants mainly encode and search for one template at a time before encoding the next template (Hoogerbrugge et al., 2023; Li et al., 2023). Participants hereby frequently resample external information which they encoded earlier – not only when searching for four complex templates, but also when searching for just one simple template (Alfandari et al., 2019; Hoogerbrugge et al., 2023). This behaviour is beneficial to completion time, accuracy, and effort; participants can spend less time and fewer VWM resources to encode templates, and instead encode or refresh VWM contents only when needed. These findings highlight the relative benefit of dynamically minimizing VWM load with the help of the external world when we can, even on simple search tasks.

The aforementioned studies have commonly considered saccades to be relatively effortless compared to VWM maintenance, but they primarily investigated the short-term dynamics of external sampling versus internal maintenance, i.e., on a trial-by-trial basis. We here instead investigated these dynamics on a more long-term scale, by examining resampling behaviour over the course of many trials. Say you are assembling a bookcase, and you need the same type of screw 25 times during the building process. In that case, it may become more time-efficient and less effortful to elaborately encode the visual search template into (long-term) memory, relative to repeatedly refreshing internal representations by making many saccades towards your instruction manual. For example, as we repeatedly search for the same target, we tend to build up an increasingly elaborate internal representation of that search target (likely in interplay with LTM; Carlisle et al., 2011; Ebbinghaus, 1885; Hout and Goldinger, 2010; Pashler et al., 2007; Woodman et al., 2007; Woodman et al., 2001). Moreover, visual search becomes relatively easy and efficient, even for multiple items, when those items are stored in LTM (e.g., Drew et al., 2017; Drew and Wolfe, 2014; Wolfe, 2012; Woodman et al., 2001, although guided search is characterised by different limitations than hybrid search). In other words, on a longer-term scale, resampling of search templates could eventually become redundant – in which case resampling behaviour should eventually cease.

We here investigated whether the preference for (re) sampling external information is persistent, and whether the balance between storing in memory versus sampling externally is different on a long-term scale than on a short-term scale. In two experiments, participants searched for templates which remained available for inspection throughout trials. Critically, each unique template set was repeated 25 times consecutively. This should make it more (effort-)efficient to elaborately encode items (either into VWM or LTM), and thus to decrease external sampling behaviour. However, given the persistence of within-trial sampling behaviour (as observed in e.g., Hoogerbrugge et al. 2023 ), it is uncertain whether participants would opt for a longer-term optimum, which may cost more effort in the short-term.

## Experiment 1

### Methods

All data together with analysis scripts and Supplementary Materials may be retrieved via the Open Science Framework https://osf.io/nr5qe/. Example videos of trials can be viewed at https://osf.io/hy9dm/. This study was not preregistered.

#### Participants and procedure

Fifteen participants (13 female, $M_{age}$ = 22.5) performed the experiment. Sample size was based on previous studies using similar paradigms (e.g., Alfandari et al. 2019; Hoogerbrugge et al. 2023)

Prior to the experiment, participants read the information letter, signed an informed consent form, and indicated their age and gender. Participants received €7 per hour or course credits, with Experiment 1 taking approximately 90 min. The study was approved by the Faculty Ethics Review Board of Utrecht University (protocol number 21-0297).

#### Apparatus

Monocular gaze location was recorded with an EyeLink 1000$+$, at 1 kHz. Stimuli were presented on a 27" 2560$\times$1440 LCD monitor at 100 Hz. Participants were seated and stabilized with a chin- and forehead rest at 67.5 cm from the monitor. The experiment was implemented with PyGaze (Dalmaijer et al., 2014).

**Fig. 1:**
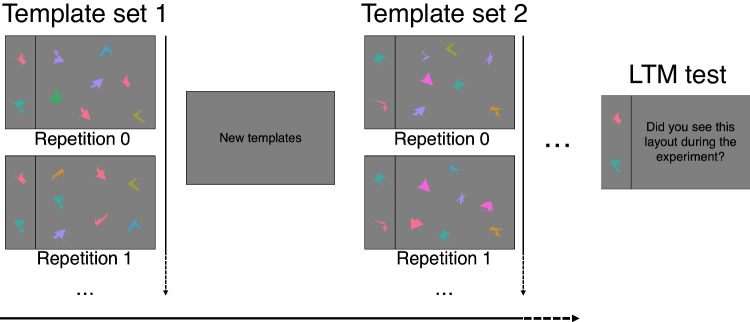
Experimental design. Participants searched whether one of the templates on the left-hand side of the vertical bar (12.7$^\circ$) was present in the search array to the right-hand side of the vertical bar (38.1$^\circ$). Participants repeated this task 25 times in a row, and search trials had no time limit. Across those 25 repetitions, the template set remained the same, and the search array was changed. After 25 repetitions, participants were shown a screen with the text "New templates". Five to ten minutes after the experiment, long-term memory of template sets was probed. Stimuli are not to scale

All gaze metrics are reported in degrees of visual angle ($^\circ$). Before the start of the experiment, and between each block, the eye tracker was calibrated and validated with a nine-dot grid, allowing a mean error of $0.5^\circ$ and a maximum per-dot error of $1.0^\circ$. The quality of calibration was automatically evaluated throughout the experiment while each pre-trial fixation cross was presented. If the gaze prediction error exceeded $1.5^\circ$ for more than two consecutive trials, the eye tracker was re-calibrated.

Fixations were detected using I2MC in Python (Hessels et al., 2017). All fixation candidates shorter than 60 ms were removed, and fixation candidates which were separated by less than $1^\circ$ distance were merged. This approach has been shown to remove variation in gaze event detection between eye trackers and fixation detection algorithms (Hooge et al., 2022).

#### Stimuli

Stimuli were a subset of complex shapes (introduced by Arnoult, 1956), which are commonly used in VWM research (e.g., Hoogerbrugge et al. 2023; Sahakian et al. 2023; Somai et al. 2020). The stimuli could be shown in four configurations ($90^\circ$ rotations) and in eight colours, equally spaced along a perceptually uniform colour map (HSLuv). One of the original 30 stimuli was removed due to its high rotational symmetry, resulting in 928 unique stimuli. Stimuli were circa $1.5^\circ$ in size.

#### Task and design

Participants performed a visual search task, in which the screen was divided into two sections by a vertical line; a smaller template area (left) and a larger search area (right; Fig. 1). The template area occupied the leftmost quarter ($12.7^\circ$) of the screen and contained either 1, 2, or 4 templates. Templates would only be shown when gaze was detected in the template area, such that participants could not peripherally attend templates and search items simultaneously. The search area occupied the rightmost three quarters ($38.1^\circ$) of the screen and contained either one target and 16 distractors in target-present trials, or no target and 17 distractors in target-absent trials. A stimulus was considered a target only if it exactly matched one of the templates (shape, colour and rotation). 75% of trials were target-present trials.

Each trial would only start if a fixation was detected at a central fixation cross. Participants indicated for each trial whether one of the stimuli in the search area matched a template or not by pressing the ’z’-key or ’/’-key, respectively. There was no time limit. Participants were instructed to be as fast and accurate as possible, and received feedback after their response (’Correct’ or ’Incorrect’ in blue or red text, respectively).

Conditions with 1, 2, and 4 templates were blocked and block order was counterbalanced according to a Latin square. Within each condition, participants searched for six unique template sets (thus resulting in 18 unique template sets in the whole experiment), each of which was repeated 25 times consecutively. Across those "repetitions", the template set remained the same, but distractors and their locations were randomly drawn without replacement. A stimulus that had previously been used as a template could not be used as a template nor as a distractor. Participants were informed about the repetitions before the experiment, and were instructed on-screen whenever a new template set was introduced. The experiment was preceded by four repetitions of two template sets as practice trials.

#### Long-term memory test

After the main body of the experiment was finished, participants were given a 5- to 10-min break. Their recognition of the template sets they had encountered during the experiment was then probed. Eighteen template sets from the experiment were presented in random order, interleaved with six foils (75%/25%).

Due to an error in the code, the true template sets could be probed multiple times – repeated occurrences of a template set were discarded from analysis (this mistake was fixed after Experiment 1). None of the actual templates could occur within the foil sets. Participants indicated whether they recognized the template sets (*yes*/*no*). Participants were informed before the start of the experiment that there would be a long-term memory test.

#### Analysis

We report four key outcome variables: (a) Gaze Crossings to Templates: the number of times that participants moved their gaze from the search area to the template area as a measure of sampling behaviour; (b) Dwell Time on Templates: the sum of all fixation durations in the template area per repetition as an indicator of how much time participants spent encoding templates (in milliseconds); (c) Response Time: the response time for each repetition, measured from trial onset until keypress (in seconds); (d) Balanced Accuracy, which takes into account the unequal balance of target-present and target-absent trials (calculated as the mean of sensitivity and specificity; 1.0 denotes perfect accuracy, 0.5 denotes chance-level accuracy; Brodersen et al., 2010).

For Gaze Crossings to Templates, we computed the mean over all six template sets per participant, per repetition. For Dwell Time on Templates and Response Time, the median was computed instead. Balanced Accuracy was also computed over all six template sets, per participant, per repetition.

For statistical analyses, we split each outcome measure into five equally sized bins (repetitions 0–4, 5–9, etc.; non-binned figures are reported in the Supplementary Figs. 1 & 3). All trials were used for analyses, including target-absent trials and incorrectly responded trials (unless stated otherwise). Analyses using only correctly answered target-present trials provided conceptually similar results.

We computed repeated-measures ANOVAs (5 bins x 3 set sizes), and report main effects of bin and template set size, as well as interaction effects between bin and set size. If the assumption of sphericity was violated for an outcome variable, we report corrected ANOVAs (Greenhouse–Geisser if $\varepsilon<$ .75, otherwise Huynh-Feldt; following Abdi, 2010).

### Results

**Fig. 2:**
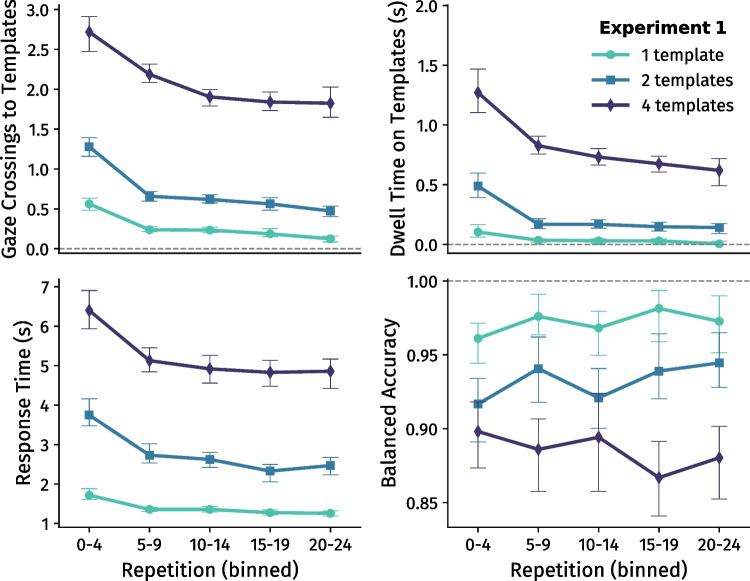
Experiment 1 outcome measures. Data were aggregated over all six template sets per participant, split per template set size and binned in sets of five repetitions. The subfigures show across-participant (*N* = 15) averages, ± 95% within-participant confidence intervals (Morey, 2008)

When a new template set was introduced, participants initially inspected the template area *M* = 1.24 (*SD* = 0.36), *M* = 1.89 (*SD* = 0.49), and *M* = 3.21 (*SD* = 1.01) times for 1, 2, and 4 templates, respectively. The number of inspections generally decreased as template sets were repeated (*F* (2.2, 30.4) = 76.07, *p* < .001, $\eta ^{2}_{p}$ = .85; Fig. 2A), and the degree of this decrease differed between set sizes: the number of gaze crossings decreased faster relative to the initial repetition for one template than for two templates, and faster for two templates than for four templates (interaction effect *F* (2.6, 36.2) = 4.21, *p* = .015, $\eta ^{2}_{p}$ = .23; Supplementary Figure 2A). Notably, participants on average still inspected the template area almost twice in the very last repetition of each template set when searching for four templates (*M* = 1.79, *SD* = 1.30). They did so in half of the final repetitions of each template set (*M* = 0.51, *SD* = 0.69) when searching for two templates – highlighting the persistence of resampling behaviour. Only when searching for one template did participants almost stop resampling in the last repetition of each template set (*M* = 0.09, *SD* = 0.14).

Similarly, the amount of time spent inspecting templates per repetition strongly dropped when a template set was first repeated, and then slowly decreased over the course of repetitions. Overall, participants dwelled longer when more templates were presented (*F* (1.2, 17.4) = 36.99, *p* < .001, $\eta ^{2}_{p}$ = .73), and dwelled shorter over the course of repetitions (*F* (1.6, 22.2) = 33.37, *p* < .001, $\eta ^{2}_{p}$ = .70; Fig. 2B). Additionally, the degree of decrease in dwell time was different per set size: dwell times decreased faster when searching for one and two templates than when searching for four templates (interaction effect *F* (2.5, 35.1) = 8.66, *p* < .001, $\eta ^{2}_{p}$ = .38; Supplementary Figure 2B).

Participants needed longer to complete the search task when searching for greater set sizes, *F* (2, 28) = 67.0, *p* < .001, $\eta ^{2}_{p}$ = .83 (Fig. 2C). Furthermore, response times decreased as template sets were repeated (*F* (2.1, 29.8) = 26.37, *p* < .001, $\eta ^{2}_{p}$ = .65), and this decrease differed between set sizes: response times decreased faster when searching for one or two templates than when searching for four templates (interaction effect *F* (4.6, 64.4) = 3.62, *p* < .001, $\eta ^{2}_{p}$ = .21; Supplementary Figure 2C).

Participants achieved higher accuracy when searching for fewer templates *F* (1.3, 18.2) = 14.07, *p* < .001, $\eta ^{2}_{p}$ = .50 (Fig. 2D), but did not get better or worse as template sets were repeated (*p* = .28), nor was there an interaction between number of templates and number of repetitions (*p* = .32).

**Fig. 3:**
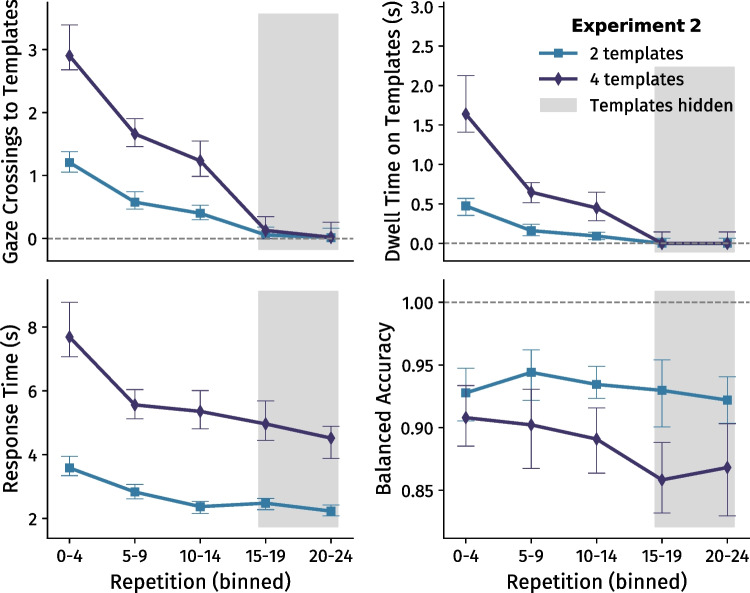
Experiment 2 outcome measures. Data were aggregated over all eight template sets per participant, split per template set size and binned in sets of five repetitions. The subfigures show across-participant (*N* = 14) averages, ± 95% within-participant confidence intervals (Morey, 2008)

### Interim discussion

Our findings show that resampling behaviour is quite persistent when visual search templates remain available. Even after 25 repetitions of the same templates, participants still inspected those templates almost twice per trial when searching for four templates, and once every two trials when searching for two templates. Only when searching for one single template did participants almost stop resampling. Over the course of those 25 repetitions, participants became quicker at completing the task, but accuracy remained stable over time.

These findings raise the question whether participants resampled templates because it helped them maintain high accuracy (i.e., template availability was *necessary* for accurate maintenance of template representations in memory). To test whether resampling was strictly necessary even after many repetitions, we ran a follow-up experiment and tested whether participants could maintain high accuracy if templates could not be inspected anymore. In Experiment 2, we allowed participants to build up memory representations by keeping templates available for inspection in the first 15 repetitions of a template set, but removed the templates for the last ten repetitions.

## Experiment 2

### Methods

Experiment 2 followed the same design, procedure, and analysis as Experiment 1, except for the following: Fourteen participants performed the experiment (12 female, $M_{age}$ = 21.6), one of which was an additional participant to replace a corrupted dataset. Participants searched for two or four templates. In each condition, eight unique template sets were presented (resulting in 16 unique template sets across the experiment), each repeated 25 times. Importantly, templates could not be inspected in the last ten repetitions of each template set. The template area was coloured a darker shade of grey in those repetitions. Participants were made aware of this before the experiment, but were not provided with the actual repetition numbers. In the long-term memory test, all 16 template sets were probed in random order, interleaved with 16 foils.

The number of gaze crossings and dwell times were analysed without taking into account the last ten repetitions, given that templates could not be inspected in those repetitions.

### Results

Similar to Experiment 1, when a new template set was introduced, participants initially inspected the template area *M* = 2.13 (*SD* = 0.81) and *M* = 4.38 (*SD* = 2.31) times for two and four templates, respectively. The number of inspections again decreased as template sets were repeated (*F* (1.2, 15.9) = 74.87, *p* $< .001, \eta ^{2}_{p}$ = .85; Fig. 3A), and the degree of this decrease differed between set sizes: the number of inspections decreased faster relative to the initial repetition when searching for two templates than when searching for four templates (interaction effect *F* (2, 26) = 14.53, *p* < .001, $\eta ^{2}_{p}$ = .53; Supplementary Figure 4A).

**Fig. 4:**
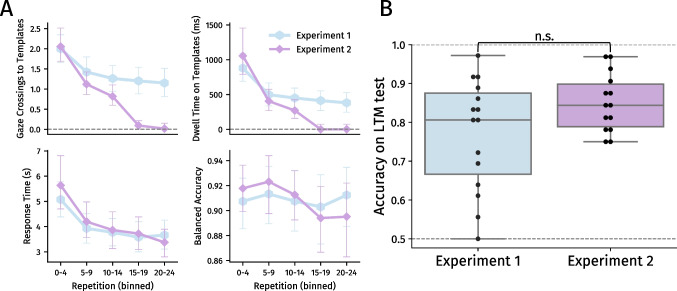
**A** Outcome measures as reported in Figs. 2 and 3, collapsed across set sizes 2 and 4, split per experiment. Repetitions were binned into groups of five to provide a clearer overview. Markers denote across-participant averages (*N* = 15 and *N* = 14, respectively), ± 95% within-participant confidence intervals (Morey, 2008). **B** Accuracy (calculated as balanced accuracy) on long-term memory tests. Accuracy of 0.5 denotes chance-level performance, 1.0 denotes perfect accuracy

Comparing between Experiments 1 and 2 (only the repetitions in which templates were available for inspection), there was an interaction effect between experiment and the number of repetitions (*F* (1.3, 36.0) = 8.85, *p* = .003, $\eta ^{2}_{p}$ = .25); participants decreased the number of inspections more quickly in Experiment 2 (Fig. 4A). Notably, however, the total number of gaze crossings made in the first 15 repetitions was similar between both experiments. When searching for two templates, participants made *M* = 12.78 (*SD* = 7.66) crossings in Experiment 1, and *M* = 10.92 (*SD* = 7.34) crossings in Experiment 2 (*t* (27) = 0.67, *p* = .51, *d* = .25). When searching for four templates, participants made *M* = 34.04 (*SD* = 15.61) crossings in Experiment 1, and *M* = 28.98 (*SD* = 12.13) crossings in Experiment 2 (*t* (27) = 0.97, *p* = .34, *d* = .36). As such, participants had overall seen templates an equal number of times in both experiments – but approached the first 15 repetitions differently in Experiment 2 than in Experiment 1.

This finding is further reflected by dwell time in the first 15 repetitions; participants initially dwelled longer than in Experiment 1but decreased their dwell times more quickly (interaction effect *F* (1.2, 32.1) = 6.76, *p* = .011, $\eta ^{2}_{p}$ = .20; Fig. 4A). However, overall, participants spent an equal amount of total time inspecting templates in the first 15 repetitions of both experiments. When searching for two templates, participants had dwelled *M* = 4.86 (*SD* = 3.11) seconds in Experiment 1, and *M* = 5.61 (*SD* = 3.47) seconds in Experiment 2 (*t* (27) = -0.61, *p* = .55, *d* = .-23). When searching for four templates, participants had dwelled *M* = 34.04 (*SD* = 15.61) seconds in Experiment 1, and *M* = 28.98 (*SD* = 12.13) seconds in Experiment 2 (*t* (27) = -0.81, *p* = .43, *d* = -.30). Within Experiment 2, the amount of time spent inspecting templates per repetition again initially dropped when a template set was repeated, and then slowly decreased further over the course of repetitions, *F* (1.2, 15.2) = 52.65, *p* < .001, $\eta ^{2}_{p}$ = .80 (Fig. 3B). Overall, there was a set size effect on dwell time (*F* (1, 13) = 25.52, *p* < .001, $\eta ^{2}_{p}$ = .66), and the degree of decrease in dwell time across repetitions was different per set size: dwell times decreased faster when searching for two templates than when searching for four templates (interaction effect *F* (1.2, 16.1) = 14.24, *p* < .001, $\eta ^{2}_{p}$ = .52; Supplementary Figure 4B).

Participants again needed more time to complete the search task when searching for greater set sizes, *F* (1, 13) = 57.39, *p* < .001, $\eta ^{2}_{p}$ = .82 (Fig. 3C). Furthermore, response times decreased as template sets were repeated (*F* (2.0, 26.2) = 24.14, *p* < .001, $\eta ^{2}_{p}$ = .65), and this decrease differed between set sizes: response times decreased faster when searching for two templates than when searching for four templates (interaction effect *F* (2.3, 29.8) = 5.0, *p* = .011, $\eta ^{2}_{p}$ = .28; Supplementary Figure 4C). Importantly, participants did not become slower or faster after template removal: There was no significant difference in response time between the last five repetitions before template removal and the repetitions thereafter, nor the final ten repetitions without templates. Comparing between the two experiments (analysing all repetitions, but only the two- and four-template conditions), there was no main effect of experiment on response time (*F* (1, 27) = 0.10, *p* = 0.749), nor were there interaction effects with template set size (*p* = .378) or the number of repetitions (*p* = .147; Fig. 4A).

Participants again achieved higher accuracy when searching for fewer templates (*F* (1, 13) = 8.0, *p* = 0.014, $\eta ^{2}_{p}$ = .38; Fig. 3D). Generally, participants did not get better or worse over time (*p* = .083). Strikingly, accuracy was not significantly different between the last five repetitions before template removal and the five repetitions after removal (e.g., 4 templates; *t* (13) = 1.70, *p* = .11, *d* = .45). To further test for the absence of a drop in accuracy after template removal, we used Bayesian ANOVAs to test for the absence of effects (BF$_{01}$). Comparing bins 2, 3, 4, and 5 (i.e., repetitions 5-24), we found evidence for the absence of a main effect of bin on accuracy; BF$_{01}$ = 2.61. Furthermore, there was moderate evidence for the absence of interaction effect between the number of templates and bin on accuracy; BF$_{01}$ = 5.99. Post hoc *t* tests further indicated weak evidence for the absence of effects between bins 3-4 (BF$_{01}$ = 1.87) and bins 3-5 (BF$_{01}$ = 2.30). Besides the former results, we tested the 4-template condition separately, since that is where the possible drop in accuracy seems most pronounced. Testing bins 2-5, again there was evidence for the absence of an effect of bin on accuracy; BF$_{01}$ = 2.51. Although these tests do not provide conclusive evidence for the absence of effects, they were consistent regardless of how the effects were tested. These findings suggest that template representations in memory were strong enough to maintain high accuracy. Furthermore, comparing between the two experiments (all repetitions; two and four templates), there was no significant effect of experiment version on accuracy (*p* = .993), nor were there interaction effects with template set size (*p* = .962) or the number of repetitions (*p* = .473; Fig. 4A).

### Interim discussion

Experiment 2 conceptually replicated the findings of Experiment 1. Although participants seemed to use a different external sampling strategy in Experiment 2 than in Experiment 1 (they sampled more early on, and decreased this more quickly), they had actually visited the template area equally frequently and for the same duration at the end of the first 15 repetitions as in Experiment 1. Together, our findings highlight that participants did not strictly need to keep resampling templates in order to boost speed or accuracy, supporting our notion that participants are persistent in their resampling behaviour, even if it does not aid standard performance metrics.

To understand why resampling was so persistent, we first examined within-trial outcomes to further elucidate whether there was indeed no short-term gain to resampling behaviour. Thereafter, we explored whether participants optimized for longer-term gain, in which case performance on long-term memory tests should improve as a result of more frequent and longer inspections during the task. Finally, we investigated whether participants resampled in order to boost metacognitive confidence rather than improve speed or accuracy.

## The purpose of search template inspections

### Short-term efficiency of template inspections

Considering a possible differentiation between short- and long-term optimization, we investigated whether there was an immediate within-trial benefit to making multiple template inspections (as observed in Hoogerbrugge et al. 2023). To analyse this, we combined data from Experiments 1 and 2 and excluded all repetitions from Experiment 2 in which templates could not be inspected. Furthermore, we excluded the first five repetitions of all template sets, given that we wanted to investigate the usefulness of resampling when templates have already been seen several times. Finally, we limited the data to trials with at most four gaze crossings in order to ensure sufficient data for analysis.

**Fig. 5:**
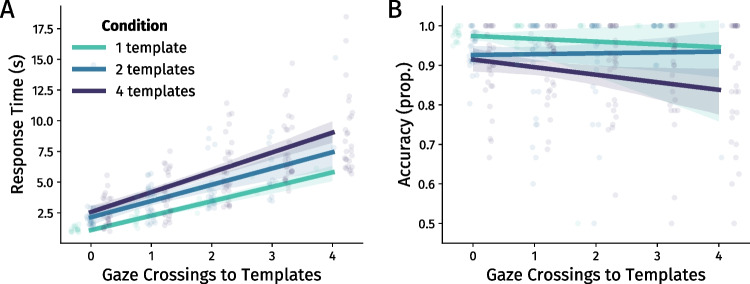
The relation between the number of gaze crossings to templates and standard performance metrics: **A** response time, **B** accuracy. *Scatterplots* show averages split by participant, template set size, and number of gaze crossings. *Solid lines* denote fitted linear regressions, with *shaded areas* around the lines denoting the 95% confidence interval. Analyses were performed using linear mixed effect models, and as such the regression lines primarily serve illustrative purposes

Linear Mixed-Effect (LME) models showed that there was a significantly positive effect of the number of gaze crossings to templates on response time, meaning that participants were generally more than a second slower at completing the task for each additional crossing they made ($\beta$ = 1.29, 95% CI [1.01, 1.59], *p* < .001; Fig. 5A; see Supplementary Materials Section 2 for a description of LMEs). Furthermore, making additional gaze crossings to the templates affected accuracy neither negatively nor positively (LME $\beta$ = -0.01, 95% CI [-0.04, 0.02], *p* = .432; Fig. 5B).

In sum, making additional template inspections was not immediately beneficial for speed. In the case of accuracy, we do not know whether a trial would have been responded to correctly if those additional crossings were not made. Therefore, additional crossings ($\ge$ 0) may have served to maintain a high level of accuracy instead of increasing it. Given that no direct within-trial benefit of resampling could be observed, we hereafter investigated possible long-term benefits.

### Long-term efficiency of template inspections

After both experiments, all participants except one could recognize templates above chance level (*M*$_{\text {exp}1}$ = 0.77, *SD* = 0.14; *M*$_{\text {exp}2}$ = 0.85, *SD* = 0.08; Fig. 4B). Thus, participants could draw at least a portion of search templates from LTM during the task – although it remains unclear whether they actually did so, or rather kept templates active in working memory.

Although average LTM accuracy was descriptively higher after Experiment 2, the difference between experiments was not significant (*t* (27) = -1.87, *p* = .073, *d* = -0.69). Therefore, having been able to inspect templates more often (Experiment 1) did not link to better or worse LTM representations than in Experiment 2, in which participants had to search without resampling for the last ten repetitions.

In order to investigate whether resampling templates was globally rather than locally optimized behaviour, we tested whether the amount and duration of inspections during the search task predicted later LTM test accuracy. We limited the data to trials with at most four gaze crossings in order to ensure sufficient data for analysis, and then compared the number of inspections made to LTM test accuracy, per template set. Participants scored better on the LTM test for larger set sizes (LME $\beta$ = 0.60, 95% CI [0.13, 1.08], *p* = .013). A greater number of inspections of a template set during the experiment was associated with *worse rather than better* recognition of that template set afterwards (LME $\beta$ = -0.63, 95% CI [-1.17, -0.09], *p* = .022). To illustrate: Template sets which were on average inspected once or less per trial were recognized with an accuracy of *M* = 0.76 (*SD* = 0.15). Inspecting template sets once up to three times was linked to similar accuracy, but with greater variance; *M* = 0.75 (*SD* = 0.26) for one-to-two crossings and *M* = 0.73 (*SD* = 0.39) for two-to-three crossings, respectively. Template sets which were on average inspected three to four times per trial were recognized worst, *M* = 0.57 (*SD* = 0.44). There was no effect of dwell time on templates.

The finding that making more inspections was linked to worse LTM recognition, and that dwell time had no effect on it, suggests that the quality of LTM representations was not a result of less elaborate encoding during the search task. Rather, it suggests that participants at least partially inspected template sets more often when they recognized that their LTM representations of those templates were worse. However, given that those additional inspections did not actually improve LTM performance, it is possible that participants may not have had the confidence to act on their template representations in memory. Therefore, these inspections may have served to boost metacognitive confidence rather than to boost the actual memory representations (in line with Desender et al. 2018; Sahakian et al. 2023).

### Template inspections for confidence boosts?

**Fig. 6:**
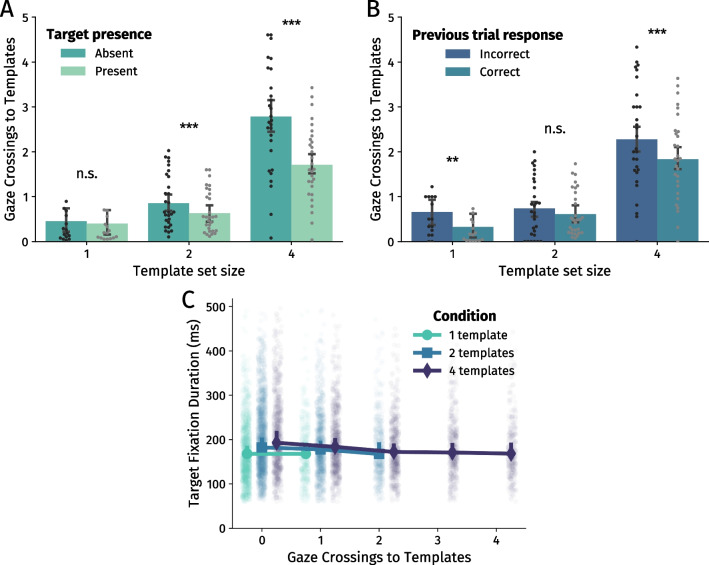
**A** Number of gaze crossings to templates, split per set size and whether trials contained a target or no target. **B** Number of gaze crossings to templates, split per set size and whether the previous trial was correct or incorrect. *Bars* in A and B denote across-participant averages (*N* = 15 for set size 1, *N* = 29 for set sizes 2 and 4), ± 95% within-participant confidence intervals (Morey, 2008). **C** Fixation duration on target, split per condition and the number of gaze crossings to templates. In order to ensure sufficient data for analysis, the number of crossings was cut-off at 1, 2, and 4 crossings for set sizes 1, 2, and 4, respectively. *Scatterpoints* show non-aggregated data (i.e., all trials without grouping). *Larger markers* denote medians over these trials, ± 95% within-participant confidence intervals. Note: paired samples *t* tests *** *p* < .001; ** *p* < .01; n.s. *p* > .05

If template re-inspections are used to boost metacognitive confidence, this should be reflected in an increased number of inspections in target-absent trials (similar to e.g., more fixations and longer response times in target-absent search; Gilchrist and Harvey, 2000; Wolfe et al., 2010). Specifically, when there is no target in the search array, one may doubt whether there was indeed no target or whether one has overlooked it. In that case, it reinspecting the templates may be a means of boosting confidence that the target was not overlooked. To this end, we combined data from both experiments (excluding the last ten repetitions from Experiment 2 in which templates were not available), split per template set size and whether trials were target-present or -absent. Participants indeed made more gaze crossings to templates in target-absent trials overall (*F* (1, 14) = 45.32, *p* < .001, $\eta ^{2}_{p}$ = .76; Fig. 6A), and did this primarily when searching for two templates (*t* (28) = 6.75, *p* < .001, Cohen’s *d* = 1.25) and when searching for four templates (*t* (28) = 9.56, *p* < .001, *d* = 1.77).

We additionally investigated behaviour which could not be caused by the experimental manipulation of target presence. Rather, we tested whether there was an increased number of inspections after giving an incorrect response, which is arguably a more natural cause of uncertainty regarding memory representations. Indeed, participants made more gaze crossings to templates after making a mistake in the previous trial, *F* (1, 14) = 28.27, *p* < .001, $\eta ^{2}_{p}$ = .67 (Fig. 6B). Specifically, participants inspected templates more frequently after a mistake when searching for one template (*t* (14) = 3.75, *p* = .002, *d* = .97) and when searching for four templates (*t* (28) = 4.54, *p* < .001, *d* = .84). Although an incorrect response could be caused by poor memory representations, we have already shown that additional inspections did not strongly aid accuracy. It is therefore likely that a large portion of inspections after errors were used to boost confidence rather than to boost memory representations.

Finally, fixation duration on search targets may reflect the internal decision-making process regarding whether a target or a distractor is fixated (e.g., Becker, 2011; Hooge and Erkelens, 1996; Wolfe, 2021; Wolfe et al. 2022) - and as such, shorter fixations on targets may indicate a higher degree of confidence. We analysed target fixation durations as a function of the number of crossings in each trial. To ensure sufficient data, we included only trials with at most 1, 2, or 4 crossings for each template set size respectively. Participants indeed fixated targets 9 ms less for each additional crossing they made towards the templates (LME $\beta$ = -9.10, 95% CI [-12.0, -6.21], *p* < .001; Fig. 6C), further supporting the notion that decision time is decreased due to higher metacognitive confidence. It should be noted, however, that participants also fixated distractors longer with each additional crossing, although by less than 2 ms (LME $\beta$ = 1.96, 95% CI [0.67, 3.24], *p* = .005).

## General discussion

When to-be-remembered information remains available for inspection throughout a trial, we often prefer to offload working memory in favour of sampling external information only when needed – largely as a means of limiting cognitive effort (Böing et al., 2023; Draschkow et al., 2021; Droll et al., 2005; Hayhoe et al., 2003; Hoogerbrugge et al., 2023; Koevoet et al., 2023a; Melnik et al., 2018; Risko & Dunn, 2015; Risko & Gilbert, 2016; Sahakian et al., 2023; Somai et al., 2020; Triesch et al., 2003). Besides limiting effort, being able to resample external information in visual search is also considered beneficial for task speed and accuracy, at least when every trial requires us to search for new templates (e.g., Hoogerbrugge et al. 2023; Li et al. 2023).

However, when we repeatedly search for the same templates, it may instead be less cognitively demanding to build strong memory representations of those templates early on, and decrease sampling behaviour as the search task repeats. In two experiments, we investigated whether resampling of external information was still the preferred strategy when participants searched for the exact same templates in 25 consecutive trials. In both experiments, when searching for a new, unfamiliar template set, participants made multiple gaze crossings towards the template area per trial – in line with aforementioned findings on short-term optimization. As template sets were repeated, participants sampled external information less, but did not actually stop sampling as long as templates remained available for inspection. In Experiment 2, we removed access to templates after 15 repetitions, and showed that participants did not strictly need to resample templates in later repetitions in terms of task speed and accuracy. Accuracy remained high when templates were made unavailable, hence our statement that resampling behaviour is *persistent*.

In the short term (i.e., within trials), more (re)sampling was associated with longer trial completion times and no benefit to accuracy. This finding deviates from previous studies (e.g., of visual search; Hoogerbrugge et al., 2023; Li et al., 2023), which introduced new templates on every trial. We therefore investigated whether resampling in our repeated search task followed a different trade-off (storing in memory versus sampling externally) than in those studies, and served long-term rather than short-term gain. Five to ten minutes after the main body of the experiment was completed, all but one participant could still recognise template sets that they had encountered at above chance level, meaning that most templates were fairly strongly represented in LTM. It should be noted that participants could have responded that they recognised a template set if they remembered only a subset of a probed set, or the inverse if they recognised none of the foils – this may therefore provide an inflated estimate of how well all templates were represented in LTM. Furthermore, merely being frequently exposed to a target can already strengthen its representation in long-term memory (Carlisle et al., 2011; Ebbinghaus, 1885; Greene & Soto, 2012; Hout & Goldinger, 2010; Pashler et al., 2007; Woodman et al., 2001, 2007), so our LTM test does not elucidate whether templates were already stored in LTM after the first few repetitions (and then consolidated through repeatedly activating those representations in memory), or if resampling in later repetitions still specifically helped to strengthen template representations. Somewhat surprisingly, we found that more resampling during the search task was actually linked to worse recognition on the LTM test, and that sampling duration did not affect this outcome. This suggests that participants may have inspected templates more frequently when their memory representations of those templates were worse, although it did not seem to help on the later test.

Evidently, the trade-off between storing internally and sampling externally is different when we know that we will have to execute the same task multiple times rather than just once. Of course, some degree of (re)sampling was necessary to (1) initially encode templates and (2) improve the memory quality of those templates or counteract memory decay, but we observed a remarkable amount of seemingly ’excessive’ sampling behaviour, given that it often did not contribute to short-term or long-term task performance.

What could then explain this behaviour? Our search task contained relatively complex stimuli (polygons which could occur in multiple rotations and colours) which may have been easy to confuse with similar items during search – although the detrimental effect of stimulus complexity on one’s ability to memorize and use them does diminish as a result of repeated exposure (Bethell-Fox & Shepard, 1988; Eng et al., 2005). Related, memory contents may be erroneous or may degrade over time (Baddeley & Hitch, 1974; Gold et al., 2005; Hardt et al., 2013; Van der Stigchel, 2020), perhaps even more so for complex stimuli. Besides limiting cognitive effort by offloading memory, resampling valid and stable external information may therefore be preferable over completely relying on ’fallible’ memory. In other words, participants may not have had the confidence to act on their template representations in memory, or increased their threshold for which level of confidence they were willing to act on. This idea is in line with Sahakian et al. (2023), who reported that participants sometimes resample external information even when there is still sufficient information in working memory, and that this depends on the ease with which external information can be accessed. Similarly, Desender et al. (2018) showed that participants regularly chose to resample external information due to low subjective confidence, even if objective task accuracy was equal. As such, we explored whether resampling behaviour was partially used to boost metacognitive confidence rather than improving the quality of memory content. Participants inspected templates more often in target-absent trials and after they had given an incorrect response, which indicates that resampling behaviour is influenced by uncertainty stemming from both current and previous trials (Gilchrist & Harvey, 2000; Wolfe et al., 2010). They also made shorter fixations on targets when they had inspected templates more often in that trial, which may indicate that the decision process was faster due to a higher degree of confidence regarding the quality of memory content (Becker, 2011; Hooge & Erkelens, 1996; Wolfe, 2021) .

Perhaps, participants spent overall more effort during the task – encoding items more actively or executing the task more attentively – when they knew that templates would become unavailable, although the effect of increased effort on improvement of memory quality is debatable (Braver et al., 2007; Master et al., 2023; Koevoet et al., 2023a; Tyler et al., 1979; Zacks et al., 1983). Alternatively, participants resampled so often because they prioritized accuracy over speed. However, due to the nature of the task, if participants truly emphasized accuracy, one would expect almost no mistakes (because they could resample ad infinitum). Accuracy in both experiments was high, but not perfect – suggesting that participants did give speeded responses. Future research may attempt to elucidate how the persistence of resampling behaviour is affected by speed-accuracy-effort trade-offs.

Making saccades towards templates may also be habitual behaviour. The template area became a darker shade of grey when templates could not be inspected in Experiment 2 – yet, participants sometimes made saccades towards the template area in the first repetition even when they were clearly unavailable (Fig. 3A), which could point to habitual or reflexive saccadic behaviour. Making a saccade is relatively effortless, and reflexive eye movements have been frequently observed in previous studies (e.g., oculomotor capture; Theeuwes, 2012; Theeuwes et al. 1998), in which involuntary saccades were often made towards novel or salient (task-irrelevant) objects, and gaze could stay at those items for up to 150 ms. In the current study, templates only appeared on screen when gaze was detected in the appropriate location, which eliminates abrupt-onset saliency as factor. Furthermore, individual fixations on templates were generally longer than 150 ms, suggesting that participants mostly made gaze crossings to the template area to actively inspect items rather than out of habit or reflex.

Participants were not told specifically after which repetition templates would become unavailable, so they would be less inclined to postpone elaborate encoding of templates until the last repetition before removal. Participants did seem to change their behaviour somewhat over the course of the experiment, as they learned to estimate when templates would be removed. In later template sets, participants sampled slightly less often and for shorter amounts of time, but the behavioural pattern remained similar: Participants frequently sampled templates when possible. Furthermore, overall response times and accuracy did not change significantly over the course of the experiment. In Supplementary Materials Section 3, we report on these possible learning effects in more detail. Moreover, the time-points at which participants inspected templates did not change over the course of trials; see Supplementary Materials Section 4.

Participants may have persistently resampled external information for various underlying reasons – i.e., due to individual differences in their working memory capacity, their willingness to load memory, willingness to utilize memory content, and in baseline metacognitive confidence. Together, our findings suggest that at least three factors play a role in the persistent resampling of external information. Besides the unavoidable initial encoding of search templates, and a regular revisit to rehearse and enhance the quality of memory representations, we showed that the boosting of metacognitive confidence is at least one of the additional reasons for this behaviour. However, this is not a comprehensive account and other factors may be at play. Moreover, we can only infer that these reasons are at play across the task but cannot estimate this for individual participants or trials.

We have here provided a novel paradigm which may provide further insights into visual search at the intersection of guided search and hybrid search paradigms. Generally, investigations into guided search employ either singletons or novel items on each trial (Liesefeld et al., 2024; Wolfe, 2021), whereas hybrid search investigates the interplay between searching through an external array and searching through (activated long-term) memory for many items (Drew & Wolfe, 2014; Drew et al., 2017; Wolfe, 2012). Our paradigm allows to investigate whether participants transition from working-memory-guided search towards a more hybrid search approach as search templates are repeated and consolidated in long-term memory. For example, after external templates were hidden in Experiment 2, participants may have suddenly switched from maintaining template representations in VWM towards retrieving those templates from LTM. In that case, the transient (but non-significant) drop in accuracy at that point potentially reflects a switch cost associated with activating representations in, or the retrieval of information from, LTM (Mayr & Kliegl, 2000; Rogers & Monsell, 1995). Moreover, response times remained stable during this period, meaning that *if* a transition between VWM and LTM indeed occurred, one is not necessarily faster than the other.

Together, our findings illustrate the persistence with which external information is resampled, even after 25 consecutive searches for the same templates. We here showed that, when eliminating the need to offload working memory, participants still resample external information, but partially to boost metacognitive confidence rather than enhancing the quality of memory representations. As such, we argue that the commonly reported trade-off between storing in memory versus just-in-time sampling externally (which is considered to be an optimization of the expenditure of cognitive effort associated with working memory maintenance) should not only be investigated in a short-term time frame, but should also take into account longer-term optimizations.

## Supplementary Information

Below is the link to the electronic supplementary material.

Supplementary file 1 (pdf 450 KB)

## Data Availability

All data and Materials may be retrieved via the Open Science Framework https://osf.io/nr5qe/. Example videos of trials can be viewed at https://osf.io/hy9dm/.

## Code Availability

All experimental code and analysis scripts and may be retrieved via the Open Science Framework https://osf.io/nr5qe/.

## References

[CR1] Abdi, H. (2010). The greenhouse-geisser correction. *Encyclopedia of Research Design,**1*(1), 544–548.

[CR2] Alfandari, D., Belopolsky, A. V., & Olivers, C. N. L. (2019). Eye movements reveal learning and information-seeking in attentional template acquisition. *Visual Cognition,**27*(5–8), 467–486. 10.1080/13506285.2019.1636918

[CR3] Arnoult, M. D. (1956). Familiarity and recognition of nonsense shapes. *Journal of Experimental Psychology,**51*(4), 269–276. 10.1037/h0047772 13306876 10.1037/h0047772

[CR4] Baddeley, A. D., & Hitch, G. (1974). *Working memory. The psychology of learning and motivation*. New York, NY: Academicp.

[CR5] Ballard, D. H., Hayhoe, M. M., & Pelz, J. B. (1995). Memory representations in natural tasks. *Journal of cognitive neuroscience,**7*(1), 66–80. 10.1162/jocn.1995.7.1.66 23961754 10.1162/jocn.1995.7.1.66

[CR6] Becker, S. I. (2011). Determinants of dwell time in visual search: Similarity or perceptual difficulty? *PLOS One,**6*(3), e17740. 10.1371/journal.pone.0017740 21408139 10.1371/journal.pone.0017740 PMC3050928

[CR7] Bethell-Fox, C. E., & Shepard, R. N. (1988). Mental rotation: Effects of stimulus complexity and familiarity. *Journal of Experimental Psychology: Human Perception and Performance,**14*, 12–23. 10.1037/0096-1523.14.1.12

[CR8] Böing, S., Ten Brink, A. F., Hoogerbrugge, A. J., Oudman, E., Postma, A., Nijboer, T. C. W., & Van der Stigchel, S. (2023). Eye movements as proxy for visual working memory usage: Increased reliance on the external world in Korsakoff syndrome. *Journal of Clinical Medicine,**12*(11), 3630. 10.3390/jcm12113630 37297825 10.3390/jcm12113630 PMC10253737

[CR9] Braver, T. S., Gray, J. R., & Burgess, G. C. (2007). Explaining the many varieties of working memory variation: Dual mechanisms of cognitive control. In *Variation in working memory* (pp. 76-106). Oxford University Press, USA

[CR10] Brodersen, K. H., Ong, C. S., Stephan, K. E., & Buhmann, J. M. (2010). The balanced accuracy and its posterior distribution. In *2010 20th International conference on pattern recognition* (pp. 3121-3124). 10.1109/ICPR.2010.764

[CR11] Carlisle, N. B., Arita, J. T., Pardo, D., & Woodman, G. F. (2011). Attentional templates in visual working memory. *Journal of Neuroscience,**31*(25), 9315–9322. 10.1523/JNEUROSCI.1097-11.2011 21697381 10.1523/JNEUROSCI.1097-11.2011 PMC3147306

[CR12] Dalmaijer, E. S., Mathôt, S., & Van der Stigchel, S. (2014). PyGaze: An open-source, cross-platform toolbox for minimal-effort programming of eyetracking experiments. *Behavior Research Methods,**46*(4), 913–921. 10.3758/s13428-013-0422-2 24258321 10.3758/s13428-013-0422-2

[CR13] Desender, K., Boldt, A., & Yeung, N. (2018). Subjective confidence predicts information seeking in decision making. *Psychological Science,**29*(5), 761–778. 10.1177/0956797617744771 29608411 10.1177/0956797617744771

[CR14] Draschkow, D., Kallmayer, M., & Nobre, A. C. (2021). When natural behavior engages working memory. *Current Biology,**31*(4), 869-874.e5. 10.1016/j.cub.2020.11.013 33278355 10.1016/j.cub.2020.11.013 PMC7902904

[CR15] Drew, T., Boettcher, S. E., & Wolfe, J. M. (2017). One visual search, many memory searches: An eye-tracking investigation of hybrid search. *Journal of Vision,**17*(11), 1–10. 10.1167/17.11.5 10.1167/17.11.5 PMC5596794 28892812

[CR16] Drew, T., & Wolfe, J. M. (2014). Hybrid search in the temporal domain: Evidence for rapid, serial logarithmic search through memory. *Attention, Perception, & Psychophysics,**76*(2), 296–303. 10.3758/s13414-013-0606-y 10.3758/s13414-013-0606-y PMC4350328 24343519

[CR17] Droll, J. A., Hayhoe, M. M., Triesch, J., & Sullivan, B. T. (2005). Task demands control acquisition and storage of visual information. *Journal of Experimental Psychology: Human Perception and Performance,**31*, 1416–1438. 10.1037/0096-1523.31.6.1416 16366799 10.1037/0096-1523.31.6.1416

[CR18] Ebbinghaus, H. (1885). Über das Gedächtnis: Untersuchungen zur experimentellen Psychologie. *Duncker & Humblot*.

[CR19] Eng, H. Y., Chen, D., & Jiang, Y. (2005). Visual working memory for simple and complex visual stimuli. *Psychonomic Bulletin & Review,**12*(6), 1127–1133. 10.3758/BF03206454 16615339 10.3758/bf03206454

[CR20] Gilchrist, I. D., & Harvey, M. (2000). Refixation frequency and memory mechanisms in visual search. *Current Biology,**10*(19), 1209–1212. 10.1016/S0960-9822(00)00729-6 11050390 10.1016/s0960-9822(00)00729-6

[CR21] Gold, J. M., Murray, R. F., Sekuler, A. B., Bennett, P. J., & Sekuler, R. (2005). Visual memory decay is deterministic. *Psychological Science,**16*(10), 769–774. 10.1111/j.1467-9280.2005.01612.x 16181438 10.1111/j.1467-9280.2005.01612.x

[CR22] Greene, C. M., & Soto, D. (2012). Neural repetition effects in the medial temporal lobe complex are modulated by previous encoding experience. *PLOS ONE,**7*(7), e40870. 10.1371/journal.pone.0040870 22829892 10.1371/journal.pone.0040870 PMC3400659

[CR23] Hardt, O., Nader, K., & Nadel, L. (2013). Decay happens: The role of active forgetting in memory. *Trends in Cognitive Sciences,**17*(3), 111–120. 10.1016/j.tics.2013.01.001 23369831 10.1016/j.tics.2013.01.001

[CR24] Hayhoe, M. M., Shrivastava, A., Mruczek, R., & Pelz, J. B. (2003). Visual memory and motor planning in a natural task. *Journal of Vision,**3*(1), 6. 10.1167/3.1.6 12678625 10.1167/3.1.6

[CR25] Hessels, R. S., Niehorster, D. C., Kemner, C., & Hooge, I. T. C. (2017). Noise-robust fixation detection in eye movement data: Identification by two-means clustering (I2MC). *Behavior Research Methods,**49*(5), 1802–1823. 10.3758/s13428-016-0822-1 27800582 10.3758/s13428-016-0822-1 PMC5628191

[CR26] Hooge, I. T. C., & Erkelens, C. J. (1996). Control of fixation duration in a simple search task. *Perception & Psychophysics,**58*(7), 969–976. 10.3758/BF03206825 8920834 10.3758/bf03206825

[CR27] Hooge, I. T., Niehorster, D. C., Nyström, M., Andersson, R., & Hessels, R. S. (2022). Fixation classification: How to merge and select fixation candidates. *Behavior Research Methods*, *2001*. 10.3758/s13428-021-01723-1 10.3758/s13428-021-01723-1 PMC9729319 35023066

[CR28] Hoogerbrugge, A. J., Strauch, C., Nijboer, T. C. W., & Van der Stigchel, S. (2023). Don’t hide the instruction manual: A dynamic trade-off between using internal and external templates during visual search. *Journal of Vision,**23*(7), 14. 10.1167/jov.23.7.14 37486300 10.1167/jov.23.7.14 PMC10382786

[CR29] Hout, M. C., & Goldinger, S. D. (2010). Learning in repeated visual search. *Attention, Perception, & Psychophysics,**72*(5), 1267–1282. 10.3758/APP.72.5.1267 10.3758/APP.72.5.1267 PMC4241378 20601709

[CR30] Kahneman, D. (1973). *Attention and effort*. Englewood Cliffs, New Jersey: Prentice-Hall Inc.

[CR31] Koevoet, D., Naber, M., Strauch, C., Somai, R. S., & Van der Stigchel, S. (2023a). Differential aspects of attention predict the depth of visual working memory encoding: Evidence from pupillometry. *Journal of Vision,**23*(6), 9. 10.1167/jov.23.6.9 37318440 10.1167/jov.23.6.9 PMC10278550

[CR32] Koevoet, D., Strauch, C., Naber, M., & Van der Stigchel, S. (2023b). The costs of paying overt and covert attention assessed with pupillometry. *Psychological Science,* 09567976231179378,. 10.1177/09567976231179378 10.1177/09567976231179378 37314425

[CR33] Li, A., Chen, Z., Wolfe, J. M., & Olivers, C. N. L. (2023). How do people find pairs?. *Journal of Experimental Psychology General*. 10.1037/xge0001390 10.1037/xge0001390 36951742

[CR34] Liesefeld, H. R., Lamy, D., Gaspelin, N., Geng, J. J., Kerzel, D., Schall, J. D.,... Wolfe, J. (2024). Terms of debate: Consensus definitions to guide the scientific discourse on visual distraction. *Attention, Perception, & Psychophysics.*[SPACE]10.3758/s13414-023-02820-3 10.3758/s13414-023-02820-3 PMC11552440 38177944

[CR35] Master, S. L., Li, S., & Curtis, C. E. (2023). *Trying harder: How cognitive effort sculpts neural representations during working memory.*[SPACE]10.1101/2023.12.07.570686 10.1523/JNEUROSCI.0060-24.2024 PMC11236589 38769009

[CR36] Mayr, U., & Kliegl, R. (2000). Task-set switching and long-term memory retrieval. *Journal of Experimental Psychology: Learning, Memory, and Cognition,**26*(5), 1124–1140. 10.1037/0278-7393.26.5.1124 11009248 10.1037//0278-7393.26.5.1124

[CR37] Melnik, A., Schüler, F., Rothkopf, C. A., & König, P. (2018). The world as an external memory: The price of saccades in a sensorimotor task. *Frontiers in behavioral neuroscience,**12*, 253. 30515084 10.3389/fnbeh.2018.00253 PMC6255858

[CR38] Morey, R. D. (2008). Confidence intervals from normalized data: A correction to Cousineau (2005). *Tutorials in Quantitative Methods for Psychology,**4*(2), 61–64. 10.20982/tqmp.04.2.p061

[CR39] Olivers, C. N., & Eimer, M. (2011). On the difference between working memory and attentional set. *Neuropsychologia,**49*(6), 1553–1558. 10.1016/j.neuropsychologia.2010.11.033 21145332 10.1016/j.neuropsychologia.2010.11.033

[CR40] O’Regan, J. K. (1992). Solving the" real" mysteries of visual perception: The world as an outside memory. *Canadian Journal of Psychology/Revue canadienne de psychologie,**46*(3), 461. 10.1037/h0084327 10.1037/h0084327 1486554

[CR41] Palmer, J., Verghese, P., & Pavel, M. (2000). The psychophysics of visual search. *Vision Research,**40*(10), 1227–1268. 10.1016/S0042-6989(99)00244-8 10788638 10.1016/s0042-6989(99)00244-8

[CR42] Pashler, H., Rohrer, D., Cepeda, N. J., & Carpenter, S. K. (2007). Enhancing learning and retarding forgetting: Choices and consequences. *Psychonomic Bulletin & Review,**14*(2), 187–193. 10.3758/BF03194050 17694899 10.3758/bf03194050

[CR43] Risko, E. F., & Dunn, T. L. (2015). Storing information in-the-world: Metacognition and cognitive offloading in a short-term memory task. *Consciousness and Cognition,**36*, 61–74. 10.1016/j.concog.2015.05.014 26092219 10.1016/j.concog.2015.05.014

[CR44] Risko, E. F., & Gilbert, S. J. (2016). Cognitive Offloading. *Trends in Cognitive Sciences,**20*(9), 676–688. 10.1016/J.TICS.2016.07.002 27542527 10.1016/j.tics.2016.07.002

[CR45] Rogers, R. D., & Monsell, S. (1995). Costs of a predictible switch between simple cognitive tasks. *Journal of Experimental Psychology: General,**124*, 207–231. 10.1037/0096-3445.124.2.207

[CR46] Sahakian, A., Gayet, S., Paffen, C. L. E., & Van der Stigchel, S. (2023). Mountains of memory in a sea of uncertainty: Sampling the external world despite useful information in visual working memory. *Cognition,**234*, 105381. 10.1016/j.cognition.2023.105381 36724621 10.1016/j.cognition.2023.105381

[CR47] Somai, R. S., Schut, M. J., & Van der Stigchel, S. (2020). Evidence for the world as an external memory: A trade-off between internal and external visual memory storage. *Cortex,**122*, 108–114. 10.1016/j.cortex.2018.12.017 30685062 10.1016/j.cortex.2018.12.017

[CR48] Theeuwes, J. (2012). Automatic Control of Visual Selection. In M. D. Dodd & J. H. Flowers (Eds.), *The Influence of Attention, Learning, and Motivation on Visual Search *(pp. 23-62). 10.1007/978-1-4614-4794-8_3 10.1007/978-1-4614-4794-8_3 23437629

[CR49] Theeuwes, J., Kramer, A. F., Hahn, S., & Irwin, D. E. (1998). Our eyes do not always go where we want them to go: Capture of the eyes by new objects. *Psychological Science,**9*(5), 379–385. 10.1111/1467-9280.00071

[CR50] Triesch, J., Ballard, D. H., Hayhoe, M. M., & Sullivan, B. T. (2003). What you see is what you need. *Journal of Vision,**3*(1), 9. 10.1167/3.1.9 10.1167/3.1.9 12678628

[CR51] Tyler, S. W., Hertel, P. T., McCallum, M. C., & Ellis, H. C. (1979). Cognitive effort and memory. *Journal of Experimental Psychology: Human Learning and Memory,**5*(6), 607–617. 10.1037/0278-7393.5.6.607

[CR52] Van der Stigchel, S. (2020). An embodied account of visual working memory. *Visual Cognition,**28*(5–8), 414–419. 10.1080/13506285.2020.1742827

[CR53] Wolfe, J. M. (2010). Visual search. *Current Biology,**20*(8). 10.1016/j.cub.2010.02.016 10.1016/j.cub.2010.02.016 PMC5678963 21749949

[CR54] Wolfe, J. M. (2012). Saved by a log: How do humans perform hybrid visual and memory search? *Psychological Science,**23*(7), 698–703. 10.1177/0956797612443968 22623508 10.1177/0956797612443968 PMC3966104

[CR55] Wolfe, J. M. (2021). Guided Search 6.0: An updated model of visual search. *Psychonomic Bulletin and Review,**28*(4), 1060–1092. 10.3758/s13423-020-01859-9 33547630 10.3758/s13423-020-01859-9 PMC8965574

[CR56] Wolfe, J. M., Kosovicheva, A., & Wolfe, B. (2022). Normal blindness: When we look but fail to see. *Trends in Cognitive Sciences,**26*(9), 809–819. 10.1016/j.tics.2022.06.006 35872002 10.1016/j.tics.2022.06.006 PMC9378609

[CR57] Wolfe, J. M., Palmer, E. M., & Horowitz, T. S. (2010). Reaction time distributions constrain models of visual search. *Vision Research,**50*(14), 1304–1311. 10.1016/j.visres.2009.11.002 19895828 10.1016/j.visres.2009.11.002 PMC2891283

[CR58] Woodman, G. F., Luck, S. J., & Schall, J. D. (2007). The role of working memory representations in the control of attention. *Cerebral Cortex,**17*(suppl–1), i118–i124. 10.1093/cercor/bhm065 17725994 10.1093/cercor/bhm065 PMC2094040

[CR59] Woodman, G. F., Vogel, E. K., & Luck, S. J. (2001). Visual search remains efficient when visual working memory is full. *Psychological Science,**12*(3), 219–224. 10.1111/1467-9280.00339 11437304 10.1111/1467-9280.00339

[CR60] Zacks, R. T., Hasher, L., Sanft, H., & Rose, K. C. (1983). Encoding effort and recall: A cautionary note. *Journal of Experimental Psychology: Learning, Memory, and Cognition,**9*(4), 747–756. 10.1037/0278-7393.9.4.747

