# Supplementary material for: Persistent resampling of external information despite 25 repetitions of the same visual search templates
Source: Atten Percept Psychophys. 2024 Sep 16;86(7):2301–14. doi: 10.3758/s13414-024-02953-z (PMC11480145; doi:10.3758/s13414-024-02953-z)
Supplement: Supplementary file 1 — (pdf 450 KB) [file 13414_2024_2953_MOESM1_ESM.pdf]

Supplementary Material to:
Persistent resampling of external information despite twenty-five repetitions of the same visual search templates

Alex J. Hoogerbrugge, Christoph Strauch, Tanja C. W. Nijboer, and Stefan Van der Stigchel

1 Supplementary Figures of Main Outcomes

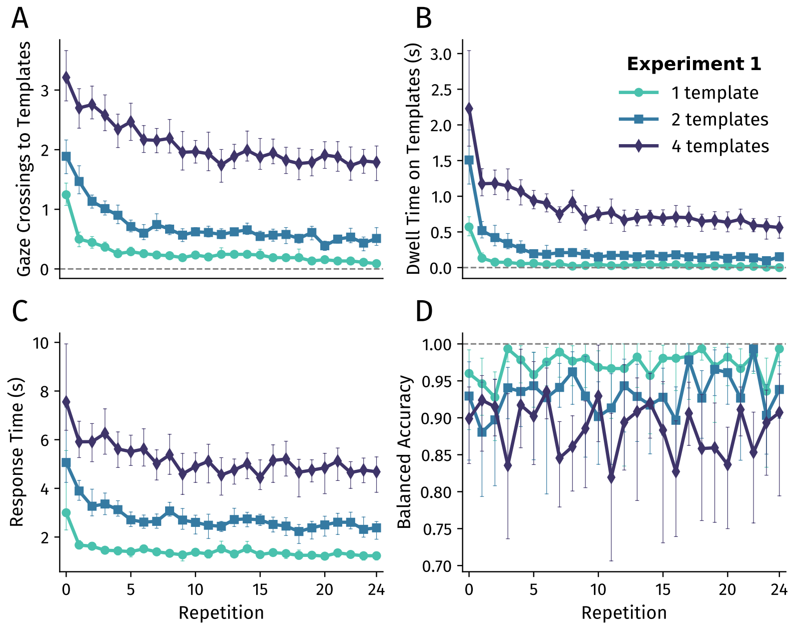

Figure 1: Experiment 1 outcome measures. Data was aggregated over all six template sets per participant, split per template set size. The subfigures show across-participant (N=15) averages, ± 95% within-participant confidence intervals (Morey, 2008).

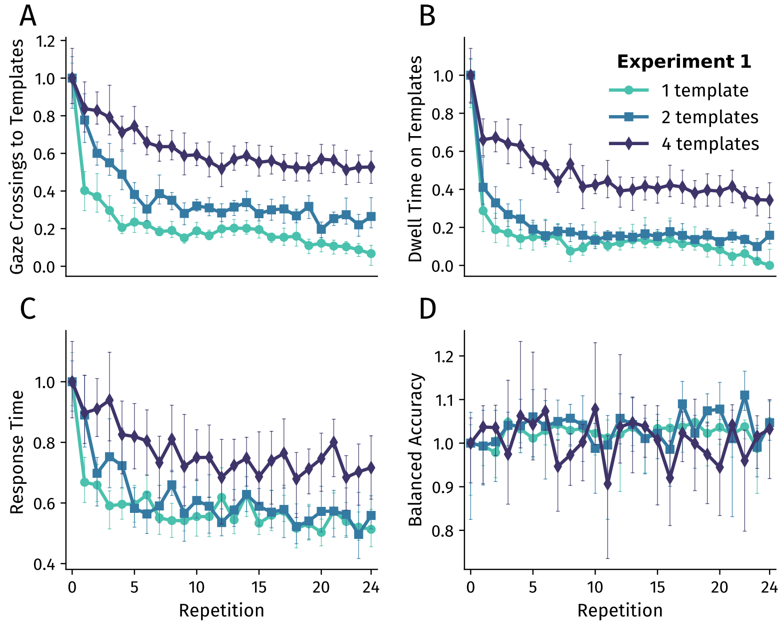

Figure 2: Experiment 1 outcome measures, relative to the initial (0th) repetition. Data was aggregated over all six template sets per participant, split per template set size and repetition. The subfigures show across-participant (N=15) averages, ± 95% within-participant confidence intervals.

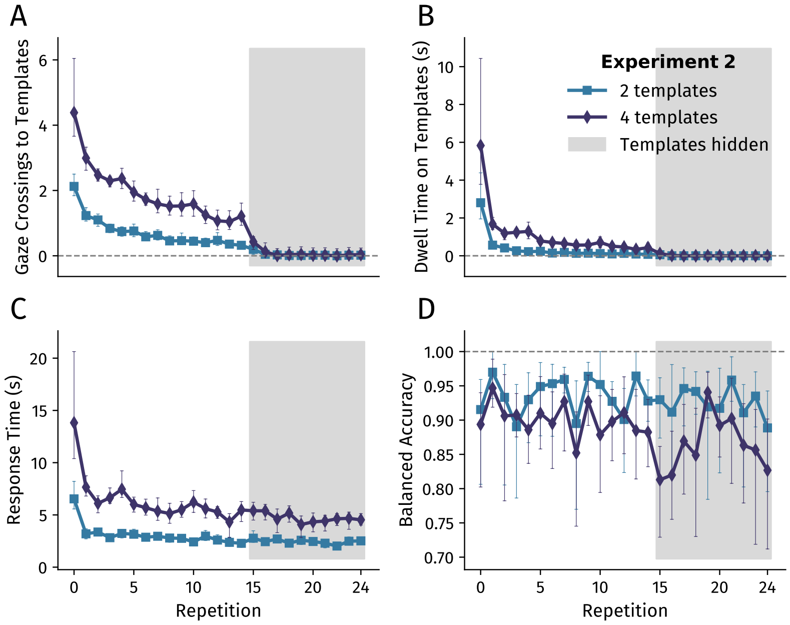

Figure 3: Experiment 2 outcome measures. Data was aggregated over all eight template sets per participant, split per template set size. The subfigures show across-participant (N=14) averages, ± 95% within-participant confidence intervals.

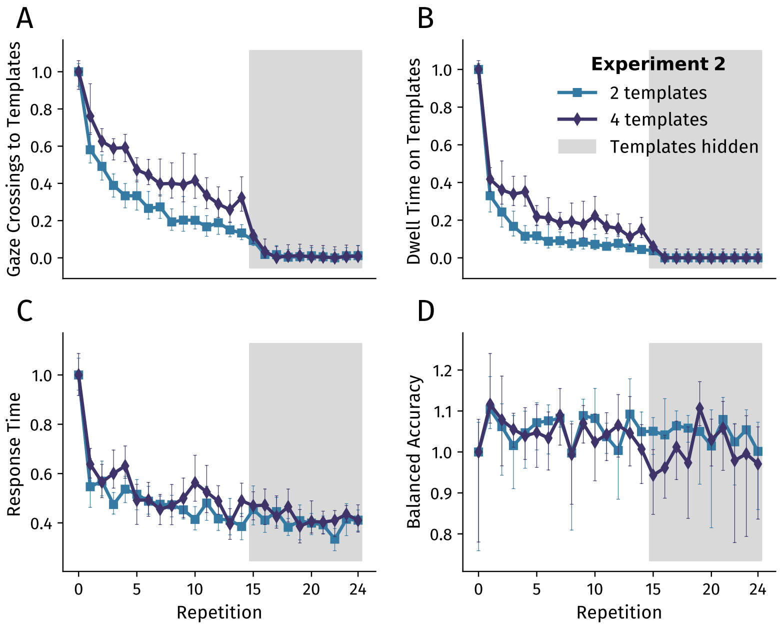

Figure 4: Experiment 2 outcome measures, relative to the initial (0th) repetition. Data was aggregated over all eight template sets per participant, split per template set size and repetition. The subfigures show across-participant (N=15) averages, ± 95% within-participant confidence intervals.

2 The purpose of search template inspections: LMEs

Linear Mixed Effect (LME) models were run with the Lmer function from lme4 (Bates et al., 2015, version 1.1-35.1) using pymer4 (Jolly, 2018, version 0.8.1). We report the formulae and output. Long-term memory scores were binary (correct or incorrect), and were therefore tested with a binomial model family. For each model, we attempted to control for as many relevant variables as possible, provided that the model would still converge. All data together with analysis scripts and supplementary materials may be retrieved via the Open Science Framework https://osf.io/nr5qe/.

2.1 Short-term benefits (paragraph 4.1)

2.1.1 Response Time

Response Time (seconds) ~ Crossings * Bin + Condition + (Crossings * Bin + Condition | ppID)

	β	2.5 % CI	97.5 % CI	*SE*	df	t	*p*	sig.
(Intercept)	0.678	0.343	1.012	0.171	22.277	3.973	0.001	***
No. crossings	1.299	1.010	1.588	0.147	12.232	8.812	< .001	***
Bin	0.056	0.010	0.101	0.023	78.224	2.369	0.020	*
Condition	0.504	0.351	0.657	0.078	27.196	6.445	< .001	***
No. crossings * Bin	0.120	-0.004	0.243	0.063	7.547	1.890	0.098	

2.1.2 Accuracy

Correct ~ Crossings * Bin + Condition + Version + (Crossings * Bin + Condition + Version | ppID)

	β	2.5 % CI	97.5 % CI	*SE*	df	t	*p*	sig.
(Intercept)	0.996	0.929	1.064	0.034	25.212	29.030	< .001	***
No. crossings	-0.013	-0.044	0.019	0.016	39.008	-0.794	0.432	
Bin	-0.002	-0.019	0.016	0.009	33.685	-0.170	0.866	
Condition	-0.020	-0.033	-0.007	0.006	30.594	-3.094	0.004	**
Version	-0.019	-0.063	0.025	0.023	19.182	-0.843	0.410	
No. crossings * Bin	-0.001	-0.011	0.010	0.005	9.101	-0.140	0.891	

2.2 LTM performance (paragraph 4.2)

LTM correct ~ Crossings + Dwell Time + Condition + (Crossings + Dwell Time + Condition | ppID); family = binomial

	β	2.5 % CI	97.5 % CI	*SE*	OR	Prob.	z	*p*	sig.
(Intercept)	0.132	-0.885	1.150	0.519	1.142	0.533	0.255	0.799	
No. crossings	-0.627	-1.165	-0.089	0.274	0.534	0.348	-2.284	0.022	*
Dwell time	0.001	-.000	0.001	.000	1.001	0.500	1.169	0.243	
Condition	0.602	0.126	1.078	0.243	1.826	0.646	2.480	0.013	*

2.3 Fixation duration (paragraph 4.3)

2.3.1 Targets

Target fixation dur. (ms) ~ Crossings + Condition + (Crossings | ppID)

	β	2.5 % CI	97.5 % CI	*SE*	df	t	*p*	sig.
(Intercept)	180.16	169.84	190.48	5.27	37.36	34.22	< .001	***
No. crossings	-9.10	-11.98	-6.21	1.47	33.13	-6.18	< .001	***
Condition	7.73	5.95	9.51	0.91	6231.61	8.51	< .001	***

2.3.2 Distractors

Distractor fixation dur. (ms) ~ Crossings + Condition + (Crossings | ppID)

	β	2.5 % CI	97.5 % CI	*SE*	df	t	*p*	sig.
(Intercept)	156.48	149.20	163.77	3.72	32.23	42.10	< .001	***
No. crossings	1.96	0.67	3.24	0.66	36.63	2.98	0.005	**
Condition	5.47	4.59	6.36	0.45	5897.14	12.10	< .001	***

3 Possible learning effects

To test for possible learning effects, we split the data based on the first three and last three template sets of each block, respectively. We used repeated-measure

ANOVAs to test for main effects of early-versus-late template sets, as well as the interaction with the number of templates and the repetition bin (Table 1 and Figure 5). Participants changed their strategy over the course of six template sets, as evidenced by two significant main effects; in later template sets they inspected templates less often and dwelled shorter. This did not affect our main conclusions, however: Although significant interaction effects between early/late template sets and repetition bin indicate that response times and accuracy were different over the course of the twenty-five repetitions, overall response times and accuracy remained unaffected by learning, as evidenced by non-significant main effects.

Table 1: Outcomes of repeated-measure ANOVAs (p-value / η_p^2) for the first three template sets (Early) versus the last three template sets (Late) of each block in Experiment 2. Tests where p<.05 are highlighted in bold font.

RM ANOVA	Gaze crossings	Dwell time	Response Time	Accuracy
Main effect of Early/Late	**.002 / .530**	**.040 / .285**	.125 / .172	.261 / .096
Templates * EarlyLate	.614 / .020	.268 / .094	.124 / .172	.415 / .052
Repetition bin * EarlyLate	**.016 / .238**	**.042 / .170**	**.017 / .203**	**.049 / .164**
Templates * Rep. bin * EarlyLate	.422 / .068	.493 / .062	.164 / .116	.731 / .038

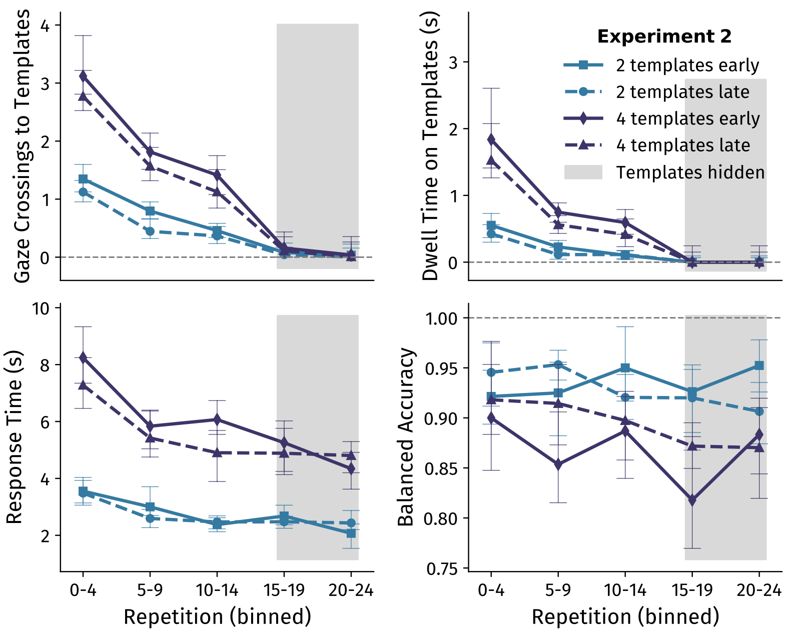

Figure 5: Experiment 2 outcome measures. Data was aggregated over all eight template sets per participant, split per template set size and binned in sets of five repetitions. Data was furthermore split between early (first 3) template sets and late (last 3) template sets per condition. The subfigures show across-participant (N=14) averages, ± 95% within-participant confidence intervals.

Furthermore, we split the data based on the counterbalanced block order; participants either performed the 2-template condition first and the 4-template condition last (Latin Square 0), or vice versa (Latin Square 1). We used mixed-design ANOVAs to test for main effects of block order, as well as the interaction with the number of templates and the repetition bin. In Figure 6, it appears that block order descriptively affected our outcome measures. However, none of the statistical tests (reported in Table 2) were significant.

Table 2: Outcomes of mixed-design ANOVAs (p-value / η_p^2). The effect of block order (Latin Square) was a between-subjects factor.

ANOVA	Gaze crossings	Dwell time	Response Time	Accuracy
Main effect of Latin Square	.467 / .045	.306 / .087	.156 / .160	.894 / .002
Templates * LS	.083 / .230	.077 / .238	.083 / .230	.745 / .009
Repetition bin * LS	.636 / .033	.602 / .054	.636 / .037	.378 / .082
Templates * Rep. bin * LS	.343 / .086	.162 / .125	.555 / .050	.123 / .138

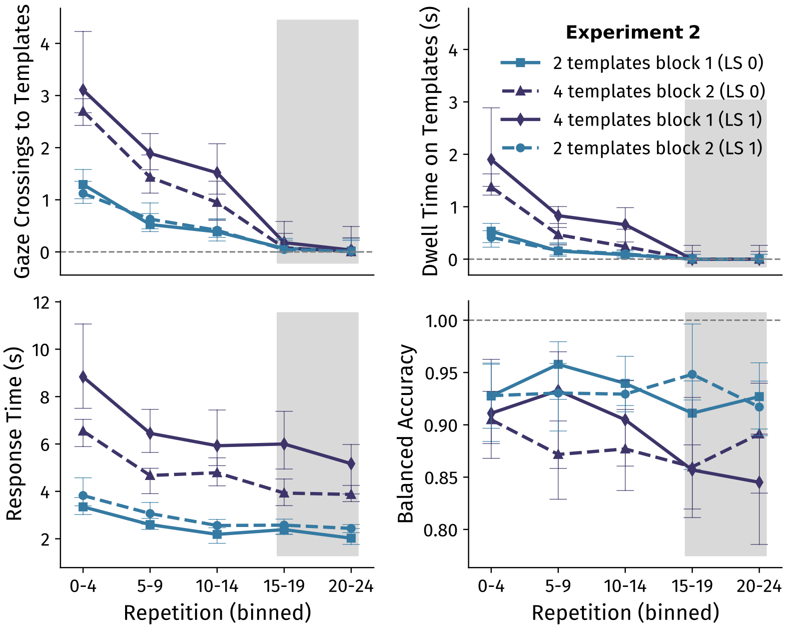

Figure 6: Experiment 2 outcome measures. Data was aggregated over all eight template sets per participant, split per template set size and binned in sets of five repetitions. Data was furthermore split based on Latin Square block order. The subfigures show across-participant (N=14) averages, ± 95% within-participant confidence intervals.

4 Timing of gaze crossings

In Hoogerbrugge et al. (2023) we analysed when crossings to templates occurred, and how often participants ended with their gaze on templates. We repeated those analyses here, though taking into account the time course of multiple repetitions.

To investigate whether there was a pattern in the timing of gaze crossings toward templates, we split our data based on the 'Nth' crossing within each trial (i.e., 1st, 2nd, . . . , 5th crossings). To retain sufficient data for analysis we only used data up to 2, 3, and 5 crossings for 1, 2, and 4 templates, respectively. However, note that the amount of data is still limited, given that many trials contained no crossings at all.

Generally, 1st crossings occurred fairly early within trials (Figure 7) – but not immediately after trial onset. If crossings occurred immediately after trial onset, we would expect to see percentages below 10%, as in Hoogerbrugge et al. (2023). This suggests that participants first searched briefly before (re)inspecting templates. Note that this data does not include trials in which no gaze crossings were made at all. The onset of 'final' crossings was generally between 60% and 80%, which is in line with our previous work (ca. 70% for both 1- and 4-template conditions). We suspect that some of this is already double-checking or 'refreshing' behaviour, as described in Hoogerbrugge et al. (2023), but participants generally still took some time (the last 20-30% of trials) to search for the double-checked item(s). Upon visual inspection, 1st crossings seemed to occur earlier in the first five repetitions than in later repetitions. Using a Linear Mixed Effect model (Table 3), we statistically tested whether crossing onsets differed between repetition bins, between Nth crossings, and between conditions. There was no main effect of the repetition bin on whether crossings occurred, nor were there significant interactions with the Nth crossing or number of templates. Thus, inspection timing was stable over the course of the 25 repetitions, which in turn suggests that participants' strategy regarding when to resample templates remained stable over time.

Table 3: LME outcomes for the onset of crossings (as a percentage of trial duration): Onset ~Bin * N^{th} crossing * No. templates + (Bin * N^{th} crossing * No. templates | ppID)

	β	2.5%CI	97.5%CI	*SE*	*df*	*T*	*p*
(Intercept)	-4.47	-21.61	12.68	8.75	55.41	-0.51	.612
Repetition bin	-1.26	-8.71	6.19	3.80	34.03	-0.33	.742
N^{th} crossing	**30.21**	**20.52**	**39.90**	**4.94**	**66.02**	**6.11**	**<.001**
No. templates	3.18	-1.58	7.94	2.43	48.75	1.31	.197
Bin * N^{th} crossing	2.56	-1.63	6.75	2.14	32.69	1.20	.239
Bin * No. templates	1.26	-0.72	3.24	1.01	29.82	1.25	.221
N^{th} crossing * No. templates	**-3.71**	**-6.19**	**-1.23**	**1.27**	**62.97**	**-2.93**	**.005**
Bin * N^{th} crossing * No. templates	-0.78	-1.84	0.28	0.54	32.15	-1.45	.157

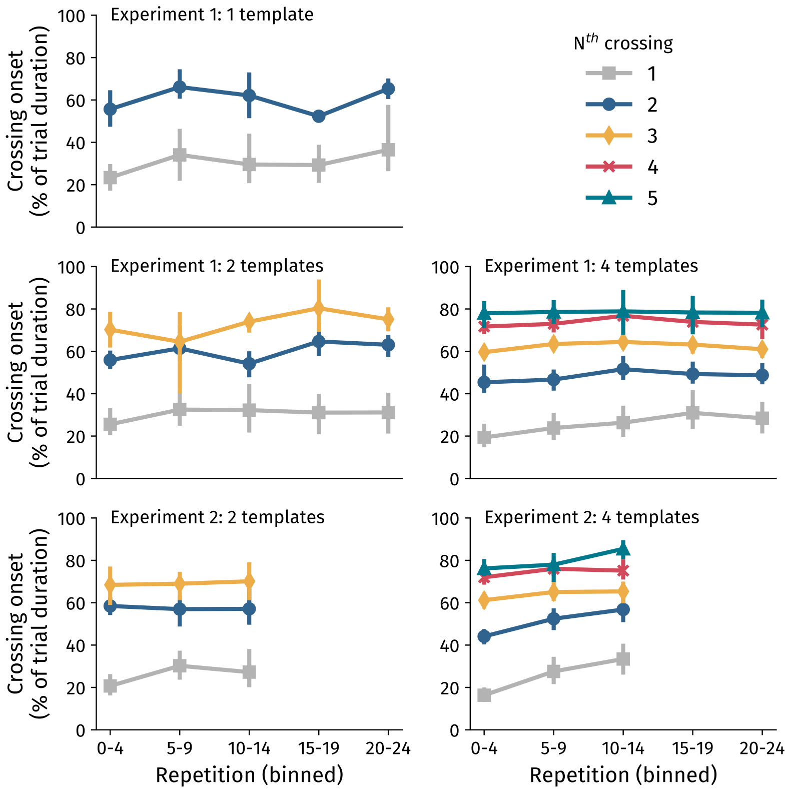

Figure 7: Onsets of crossings towards the template area as a percentage of trial duration, across repetition bins. Split by N^{th} crossing and by condition. Points denote averages, errorbars denote $\pm$ 95% within-participant confidence intervals.

Furthermore, in our previous work, we found that participants ended with their gaze on templates in 5-10% of trials on average (ranging between 0% and 35%; Figure 5B in Hoogerbrugge et al. (2023)). Moreover, ending a trial while inspecting templates was linked to higher accuracy. In the current study, we found that trials in which gaze ended on the templates occurred considerably less often overall (Figure 8A). In order to obtain a closer comparison to our previous work – in which each trial contained new templates and therefore at least one crossing was always necessary – we included only trials in which any crossings were made at all (Figure 8B). The percentage of trials in which gaze ended on templates was still lower than in Hoogerbrugge et al. (2023). Finally, we split this data based on whether a correct or incorrect response was given after 'double-checking' (Figure 8C). From this, there is no evidence that double-checking at the end of trials was linked to a benefit for accuracy – but the number of occurrences is small, thus we do not wish to over-interpret this outcome measure.

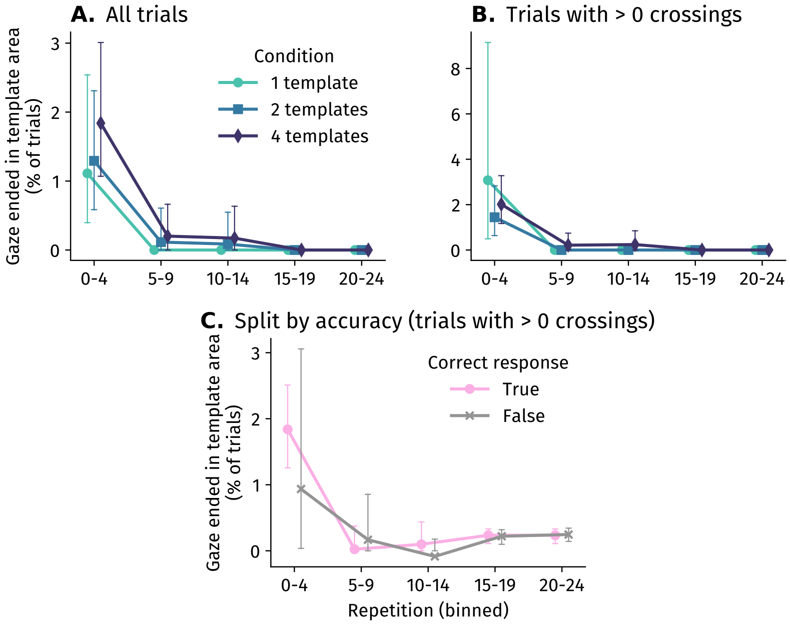

Figure 8: **A.** Percentage of trials in which gaze ended in the template area, split per condition. Points denote across-participant averages $\pm$ 95% within-participant confidence intervals. **B.** Percentage of trials in which gaze ended in the template area, split per condition. Only for trials in which more than zero crossings were made. Points denote across-participant averages $\pm$ 95% within-participant confidence intervals. **C.** Percentage of trials in which gaze ended in the template area, aggregated over conditions, split by correct/incorrect response. Only for trials in which more than zero crossings were made. Points denote across-participant averages $\pm$ 95% within-participant confidence intervals.

There is a possible explanation for the discrepancy with Hoogerbrugge et al. (2023). There, participants would encode templates, then search, then after searching decide whether a double check was necessary. In the current study however, participants could already decide soon after trial onset (or even in between trials) whether their memory representations were 'good enough' to start searching. Thus, double checks at the end of trials were probably less beneficial than in our previous study – again emphasizing the need to study resampling behaviour in the context of longer-term optimizations.

References

Bates, D., Mächler, M., Bolker, B., & Walker, S. (2015). Fitting Linear Mixed-Effects Models Using lme4. *Journal of Statistical Software*, *67*, 1–48. https://doi.org/10.18637/jss.v067.i01

Hoogerbrugge, A. J., Strauch, C., Nijboer, T. C. W., & Van der Stigchel, S. (2023). Don't hide the instruction manual: A dynamic trade-off between using internal and external templates during visual search. *Journal of Vision*, *23*(7), 14. https://doi.org/10.1167/jov.23.7.14

Jolly, E. (2018). Pymer4: Connecting R and Python for Linear Mixed Modeling. *Journal of Open Source Software*, *3*(31), 862. https://doi.org/10.21105/joss.00862

Morey, R. D. (2008). Confidence Intervals from Normalized Data: A correction to Cousineau (2005). *Tutorials in Quantitative Methods for Psychology*, *4*(2), 61–64. https://doi.org/10.20982/tqmp.04.2.p061
